# Completeness of patient-held records: observations of the Road-to-Health Booklet from two national facility-based surveys at 6 weeks postpartum, South Africa

**DOI:** 10.7189/jogh.08.020901

**Published:** 2018-12

**Authors:** Trisha Ramraj, Ameena E Goga, Anna Larsen, Vundli Ramokolo, Sanjana Bhardwaj, Witness Chirinda, Debra Jackson, Duduzile Nsibande, Kassahun Ayalew, Yogan Pillay, Carl J Lombard, Nobubelo K Ngandu, for the South Africa PMTCT Evaluation (SAPMCTE) Team

**Affiliations:** 1 Health Systems Research Unit, South African Medical Research Council, Cape Town, South Africa; 2 Department of Paediatrics and Child Health, Kalafong Hospital, University of Pretoria, South Africa; 3 US Centers for Disease Control and Prevention, Center for Global Health, Division of Global HIV and Tuberculosis, Pretoria, South Africa; 4 UNICEF, Pretoria, South Africa; 5 School of Public Health, University of the Western Cape, Cape Town, South Africa; 6 UNICEF, New York, New York, USA; 7 National Department of Health, Pretoria, South Africa; 8 Biostatistics Unit, South African Medical Research Council, Cape Town, South Africa; 9 School of Public Health and Family Medicine, University of Cape Town, South Africa

## Abstract

**Background:**

Continuity of care is important for child well-being in all settings where postnatal retention of mother-infant pairs in care remains a challenge. This analysis reports on completeness of patient-held infant Road to Health Booklets (RtHBs), amongst HIV exposed and unexposed infants during the first two years after the RtHB was launched country-wide in South Africa.

**Methods:**

Secondary data were analysed from two nationally representative, cross-sectional surveys, conducted in 2011-12 and 2012-13. These surveys aimed to measure early effectiveness of the national programme for preventing vertical HIV transmission. Participants were eligible for this analysis if they were 4-8 weeks old, receiving their six-week immunisation, not needing emergency care and had their RtHBs reviewed. Caregivers were interviewed and data abstracted from RtHBs. RtHB completeness across both surveys was defined as the proportion of RtHBs with any of the following indicators recorded: infant birth weight, BCG immunisation, maternal syphilis results and maternal HIV status. A partial proportional odds logistic regression model was used to identify factors associated with completeness. Survey sampling weights were included in all analyses.

**Results:**

Data from 10 415 (99.6%) participants in 2011-12 and 9529 (99.2%) in 2012-13 were analysed. Overall, recording of all four indicators increased from 23.1% (95% confidence interval (CI)  = 22.2-24.0) in 2011-12 to 43.3% (95% CI = 42.3-44.4) in 2012-13. In multivariable models, expected RtHB completeness (ie, recording all four indicators vs recording of <4 indicators), was significantly (*P*<0.05) associated with survey year, marital status, socio-economic status, maternal antenatal TB screening, antenatal infant feeding counselling, delivery at a clinic or hospital and type of birth attendant.

**Conclusions:**

Routine patient-held infant health RtHB, a critical tool for continuity of care in high HIV/TB prevalence settings, was poorly completed, with less than 50% of the RtHB showing expected completeness. However, government efforts for improved usage of the booklet were evidenced by the near doubling of completeness from 2011 to 2013. Education about its importance and interventions aiming at optimising its use without violating user privacy should be continued.

Since 1994, South Africa has made considerable progress in improving the health status of children. With children under the age of 5 years representing approximately 11% of the South African population, national child health policies have focused specifically on the improvement of infant and child health and nutrition [1-2]. Although, improvements are evident in the reduction of key indicators such as the infant mortality rate from 38 to 34 per 1000 live births and the under 5 mortality rate from 54 to 41 per 1000 live births between 2010 and 2015, a large proportion of these deaths were preventable [1,3,4]. Tracking new-born infants until the age of five years is critical for reducing morbidity and mortality and improving health and development outcomes of children.

Loss to follow up of infants along child health programmes such as the immunization or the prevention of mother-to-child transmission of HIV (PMTCT) cascade renders continuity of care post-delivery difficult. Missed opportunities for early infant diagnosis of HIV status or initiation of antiretroviral treatment are often attributed to poor recording of mother’s and/or the infant’s HIV status on patient-held infant health records such as the Road to Health Book (RtHB), poor maternal knowledge about mother-to-child transmission of HIV (MTCT) and the absence of provider-initiated counselling and testing service for the undocumented, unknown, or undeclared HIV-exposed infants [5]. The South African PMTCT programme stipulates that every contact between the child and the health care service should be an opportunity to check the RtHB and to determine the child’s HIV-exposure status [6]. Following routine HIV testing of HIV exposed infants at birth and 10 weeks postpartum, every child’s HIV-exposure status should be determined at monthly well baby visits. HIV testing should be repeated at any point if the child is symptomatic and the results obtained together with the laboratory tracking barcode must be documented on the RtHB).

Key to the provision of effective and efficient health care inter alia is the ability to track and retain patients in care through a well-functioning health information system. When mothers and children move from one clinic to another or even from one service to the next within the same clinic, patient-level data (for example HIV-exposure status and HIV test results) required to deliver the necessary longitudinal health care is often of poor quality, or is unrecorded or lost [7].

Currently, the RtHB is the only, readily available tool to be used for monitoring child growth, providing key child health and nutrition messages and tracking uptake of child health care services in South Africa. The patient-held RtHB provided to all children at birth is a simple tool for monitoring and managing these services. The RtHB used in South Africa has been updated four times with the last update being in 2010 [7]. The current South African RtHB replaced the Road to Health Card (RtHC) in 2010 and was fully launched country-wide by the National Department of Health in 2011 [8]. Unlike the RtHC, the RtHB included designated spaces for recording PMTCT- and HIV-related information of mother and infant. Prior to this new RtHB, the recording of PMTCT- and HIV-related information was optional, codes were used to document maternal HIV status but no designated spaces existed for recording such information. The new RtHB thus systematically institutionalises recording of vital information, including infant HIV exposure, immunizations, exposure to PMTCT interventions, Vitamin A supplementation and growth monitoring (including the weight-for-age, height-for-age, weight-for-height Z-score charts and mid-upper arm circumference) [9,10].

Preliminary analyses of data from the nationally representative South African PMTCT evaluation (SAPMTCTE) demonstrated that the absence of documentation of maternal HIV status on the RtHB was associated with a significantly increased risk of postnatal MTCT, and MTCT-or-death (personal communication, A Goga). Given these data, there is a dire need to understand and evaluate optimal use of the RtHB across the country. Using data from two large nationally representative SAPMTCTEs, this paper aims to report on the average completeness of the RtHB during the first 6 weeks postpartum and to understand factors that hinder or enable its optimal use. It will benefit future attempts to promote optimal use of the booklet and clarify our understanding as to whether RtHB incompleteness is largely a provider-related problem or certain maternal factors also play a role.

## METHODS

### Study design

Data from two nationally-representative cross-sectional South African PMTCT surveys conducted in 2011-12 (August 2011 – March 2012) and 2012-13 (October 2012 – May 2013) were used. These surveys were designed primarily to measure MTCT by 6 weeks postpartum. The surveys enrolled mother/caregiver-infant pairs at public primary health care facilities (PHCs) and community health centres (CHCs) offering immunization services. A multi-stage (at provincial, health facility and individual levels), stratified cluster sampling approach was used. Facility size was measured using expected number of DTP (diphtheria, tetanus, and pertussis) immunisations at 6 weeks. The strata were then created by combining the determined size with known maternal HIV prevalence (categorised into either high or low relative to the national average). Sample size at provincial and facility levels were then determined using probability proportionate to size with a target of providing reasonable provincial and national level estimates. Details of the primary studies have been published elsewhere [11,12]. Ethical approval for the study was granted by the South African Medical Research Council’s ethics committee and provincial research ethics committees. The protocol was also approved by the United States Centers for Disease Control and Prevention (Atlanta Georgia, USA) Center for Global Health Associate Director for Science.

### Data collection

In each of the primary studies, trained data collectors recruited eligible mother/caregiver infant pairs consecutively or systematically, depending on facility size, until the targeted sample size was achieved. Infants aged 4-8 weeks who were receiving their six-week immunisation on the day of visit and who were not in need of emergency care were eligible for inclusion in the study following signed informed consent (administered in the participant’s preferred language). Face-to-face interviews were conducted after the routine visit activity was completed and data were captured electronically using mobile phones and then stored in an access-restricted database. Data collected through interviews included maternal socio-demographic backgrounds, antenatal care histories and early postnatal health care uptake.

All enrolled participants were asked to present the RtHB during the study interview. During the survey interviews, data were extracted from the RtHB, including infant birth weight, BCG immunization, infant HIV exposure status and maternal syphilis testing results. These data are from the earliest two years after the full country-wide implementation of the RtHB (and phasing out of the RtHC) to which PMTCT indicators had been added.

### Variables

#### Outcome variable

The primary outcome used is “completeness of the RtHB”. Therefore, the data for this study were restricted to the sample of enrolled participants who brought the RtHB during the study interview and these were N = 10 415 (99.6%) in 2011-12 and N = 9529 (99.2%) in 2012-13. We created a composite outcome variable using extracted data for four variables that should have been completed at birth, namely; infant birth weight, BCG immunization, maternal HIV status and indication of whether maternal syphilis testing was done. Therefore, the outcome variable is ordinal with counts from 0 through to 4. These indicators were simply chosen because they were of common interest to both survey aims and are also important to assess PMTCT and maternal and child health service uptake.

#### Independent variables

Maternal characteristics potentially associated with uptake of health care services were assessed for association with completeness of the RtHB. These were age, educational qualification, marital status, parity, knowledge of MTCT modes and relative socioeconomic status (SES). Participants were defined as having good knowledge of MTCT if they understood the definition of MTCT and could correctly identify all three modes of MTCT viz. transmission during pregnancy, childbirth and breastfeeding, whilst those who did not understand the definition of MTCT or were not able to identify all modes were defined as having poor knowledge of MTCT. SES, grouped into quartiles, was calculated for each year using principal component analyses from household characteristics (which included type of housing, sanitation, water and fuel), household possessions (such as TV, stove, radio), any food shortage and source of income [13]. Differences between provinces and survey years were also evaluated in relation to the primary outcome. Variables which indirectly reflect competence of health service provision before 6 weeks postpartum were also included to give an indication of whether incompleteness of the booklet is related to the service provider. These variables were receipt of TB screening during pregnancy, receipt of infant feeding counselling during pregnancy, place of delivery (hospital, clinic or home) and type of birth attendant (doctor, nurse/midwife/community health worker or traditional birth attendants). We hypothesize that performance of health care providers in providing basic services recommended during pregnancy, would reflect their diligence is recording patient-held records, including the RtHB when it is issued.

### Statistical analysis

Data analysis was done in STATA version SE 13 (Stata Corp, College Station, TX, USA). An ordered logistic regression analyses was used for the outcome variables. Survey sampling weights were used to account for the sample design (ie, multi-stage & strata size proportionality) and realisation (ie, adjustment for attained vs target sample size within each stratum).

An additional subcategory was created for independent variables which had more than 5% of data entries in each survey year missing or with ‘don’t know’ responses to the questionnaire. This was done to minimise deviation from the correct estimates when applying the survey sampling weights which were calculated based on the actual attained sample size. The sub-category for ‘unknown’ responses was created for TB screening during delivery and for knowledge of MTCT modes.

Factors associated with completeness of the RtHB were identified using a partial proportional odds (PPO) logistic regression analyses in three steps [14,15]. The assumption of proportionality of odds across sub-group pairs of the outcome variable, made by the proportional odds logistic regression model, was first tested for all predictor variables using the Brant test.

The Brant test output for proportionality of odds across the categories of the outcome variable for each predictor variable are presented as χ^2^ statistics and probability values for the null hypothesis that the odds of a predictor variable are proportional across the different binary groupings of the outcome variable (see Table S1 in **Online Supplementary Document(Online Supplementary Document)**). The possible ordered binary groupings were; completeness ≥1 vs <1, completeness ≥2 vs <2, completeness ≥3 vs <3 and completeness = 4 vs <4 recorded variables. The proportionality of odds assumption was supported in four predictor variables (mother’s education, marital status, parity and birth attendant) and hence these were constrained accordingly in the regression analyses. The rest of the predictor variables violated the proportional odds model assumption and thus were not constrained under this assumption.

The PPO model was then used with a function to only constrain proportionality to those predictor variables which were consistent with the parallel regression assumptions (Brant test p-values ≥0.05) and leaving the rest unconstrained. In step 1, separate bivariate PPO logistic regression analyses taking into account the proportionality test results and constraining/not constraining accordingly, were done between the outcome and each predictor variable (estimates ‘unadjusted’ for possible confounding by other variables). In step 2, those with Wald’s test *P*-value <0.25 in step 1, indicating potential influence of the predictor on changes in the outcome, were included into a multivariable PPO logistic regression model (to get ‘adjusted’ estimates). In step 3, those predictor variables not included in step 2 were then included, one at a time, and if they significantly shifted the estimates of any predictor variable already in the multivariable model, by shifting the result for proportional odds assumption or the 95% confidence intervals (CI) of the odds ratio completely, then they were retained in the final model, otherwise they were not included.

A χ^2^ test was used to present descriptive summaries of; independent variables by survey year; completeness of each of the 4 health indicators in the RtHB by survey year; and expected completeness (all 4 indicators recorded in the RtHB) by each independent variable.

## Results

Out of all those eligible and consenting for full participation in the study, 10 415 in 2011-12 and 9529 in 2012-13 were in possession of a RtHB during the study interview.

### Socio-demographic profile (and independent variables) of the population

Across both surveys, infants were generally brought to the clinic by their mothers (97%). The majority of mothers were between the ages of 20-34 years with a nearly 2% significant decrease in teenage mothers over time, had an education level of high school or above and the majority of mothers described themselves as either single, widowed, divorced or separated, as shown in **Table 1**. More than 50% of mothers were multiparous and reported that they were HIV-negative before delivery. Self-reported antenatal maternal HIV-positive status increased between the two surveys (30.2% (95% confidence interval CI = 29.1-31.3) in 2011-12 and 32.0% (95% CI = 31.0-33.1) in 2012-13, *P* = 0.065). Knowledge of MTCT modes was around 60% in both 2011-12 and 2012-13 but appeared to be significantly lower by 2% points in 2012-13 (*P* = 0.0006).

**Table 1:** Socio-demographic profile of population and other independent factors enrolled in the 2011-12 and 2012-13 surveys, South Africa

Characteristics	Categories	2011-12	2012-13	*P*-value
**Sample size**		**N = 10 415**	**N = 9529**
**Age (years)**	13-19	16.8 (16.0-17.6)	15.0 (14.2-15.8)	0.018
	20-24	30.2 (29.2-31.2)	30.9 (29.9-32.0)	
	25-34	40.8 (39.8-41.9)	42.0 (41.0-43.1)	
	35-50	12.2 (11.5-12.9)	12.0 (11.3-12.7)	
**Mother’s education (%)**	Grade 7 and below	14.7 (14.0-15.5)	14.6 (13.9-15.4)	0.893
	Grade 8 and above	85.3 (84.5-86.0)	85.4 (84.6-86.1)	
**Marital status (%)**	Single/Widowed/Divorced/Separated	74.9 (74.0-75.8)	76.2 (75.3-77.1)	0.199
	Married/Co-habiting	25.1 (24.2-26.0)	23.8 (22.9-24.7)	
**Parity (%)**	First child	39.2 (38.1-40.2)	38.0 (37.0-39.1)	0.161
	Child 2+	60.8 (59.8-61.9)	62.0 (60.9-63.0)	
Maternal reported HIV status before delivery* (%)	HIV-	69.8 (68.7-70.9)	68.0 (66.9-69.0)	0.065
	HIV+	30.2 (29.1-31.3)	32.0 (31.0-33.1)	
Correct knowledge of MTCT modes* (%)	Doesn’t know MTCT/Doesn’t know all modes correctly	31.4 (30.4-32.4)	36.7 (35.6-37.7)	0.0006
	Knows all 3 modes correctly	60.8 (59.8-61.8)	58.5 (57.4-59.6)	
	Response unknown	7.8 (7.3-8.4)	4.8 (4.4-5.3)	
**SES (%)**	highest	27.3 (26.3-28.3)	26.1 (25.2-27.1)	0. 480
	Second highest	23.8 (22.9-24.7)	23.7 (22.8-24.7)	
	Low	23.4 (22.5-24.3)	24.7 (23.8-25.6)	
	Lowest	25.6 (24.6-26.5)	25.5 (24.6-26.4)	
**TB screening during pregnancy (%)**	No	56.4 (55.4-57.5)	59.5 (58.4-60.6)	0. 117
	Yes	36.7 (35.7-37.8)	34.1 (33.1-35.2)	
	Response Unknown	6.9 (6.4-7.4)	6.4 (5.9-7.0)	
**Infant feeding counselling during pregnancy (%)**	No	10.1 (9.4-10.7)	7.5 (7.0-8.1)	0.001
	Yes	89.9 (89.3-90.6)	92.5 (91.9-93.0)	
**Place of delivery (%)**	Hospital	79.5 (78.6-80.4)	78.6 (77.7-79.5)	0.004
	Clinic	15.9 (15.1-16.7)	17.9 (17.1-18.8)	
	Home/other	5.6 (4.1 - 7.0)	3.5 (3.1 - 3.9)	
**Birth attendant (%)**	Doctor	27.3 (26.3-28.3)	29.1 (28.1-30.1)	0.0007
	Nurse/midwife/CHW	68.4 (67.4-69.5)	67.8 (66.8-68.8)	
	TBA/other	4.3 (3.8-4.8)	3.1 (2.7-3.5)	
**Province (%)**	EC	11.2 (10.6-11.8)	11.1 (10.5-11.8)	0.249
	FS	4.7 (4.4-5.0)	5.6 (5.2-5.9)	
	GP	25.8 (24.8-26.8)	22.9 (22.0-23.9)	
	KZN	21.2 (20.2-22.2)	21.8 (20.8-22.9)	
	LP	11.5 (10.8-12.1)	11.6 (11.0-12.2)	
	MP	7.6 (7.2-8.1)	8.1 (7.6-8.6)	
	NC	2.0 (1.9-2.2)	2.3 (2.1-2.5)	
	NW	6.8 (6.4-7.3)	7.3 (6.9-7.8)	
	WC	9.1 (8.6-9.6)	9.3 (8.8-9.8)	

CHW – community health worker, MTCT – Mother-to-Child Transmission, SES – Socioeconomic Status, TB – Tuberculosis, TBA – Traditional Birth Attendant, FS – Free State, GP – Gauteng, KZN – KwaZulu-Natal, LP – Limpopo, MP – Mpumalanga, NC – Northern Cape, NW – North West, WC - Western Cape

*Category for missing data/unknown responses added. The *P*-value is from a χ^2^ test.

The proportion of women reporting to have been screened for TB during pregnancy was very low, below 40% across both survey years. On the other hand, most women had received infant feeding counselling during pregnancy, with a significant increase from 89.9% in 2011-12 to 92.5% by 2012-13 (*P* = 0.001). In both survey years, just more than 75% of child deliveries took place in hospitals, less than 20% at a clinic while home deliveries significantly decreased from 5.6% in 2011-12 to 3.5% in 2012-13 (*P* = 0.004). Approximately two thirds of deliveries were attended by nurses, midwives or community health workers. Doctor-facilitated deliveries increased significantly by nearly 2% between 2011-12 (27.3%) and 2012-13 (29.1%) while deliveries by traditional birth attendants decreased significantly from 4.3% to 3.1% during the same period (*P* = 0.0007).

Significant differences in the distribution of variables between years were evident for maternal age, knowledge of MTCT modes, infant feeding counselling, place of delivery and birth attendant.

### Completeness of the RtHB

Recording of each of the four health indicators in the RtHB were compared between the survey years (**Table 2**). With the exception of infant BCG immunisation, there was a significant increase in the recording of the remaining three indicators on the RtHB with the most notable increase in the recording of infant birth weight (67.2% (95% CI = 66.2-68.2) in 2011-12 to 89.8% (95% CI = 89.1-90.4) in 2012-13, *P* < 0.0001) and the recording of maternal HIV status (49.1% (95% CI = 48.0-50.2) in 2011-12 to 67.8% (95%C = 66.8-68.8) in 2012-13, *P* < 0.0001). By the 2012-13 survey recording of infant birth weight and infant BCG immunisation was >89% whilst recording of maternal HIV outcome and syphilis results remained below 70%.

**Table 2:** Summary of completeness of the RtHB in the 2011-12 and 2012-13 surveys, South Africa

	2011-12, % (95% CI)		2012-13, % (95% CI)		*P*-value	
	**(N = 10 415)**		**(N = 9529)**	
**Are following recorded on RtHB:**						
Infant birth weight		67.2 (66.2-68.2)		89.8 (89.1-90.4)		<0.0001
Infant BCG immunisation		99.1 (98.9-99.3)		97.5 (97.1-97.8)		0.0001
Syphilis screening results		63.6 (62.5-64.6)		69.7 (68.7-70.7)		0.0004
Maternal HIV status		49.1 (48.0-50.2)		67.8 (66.8-68.8)		<0.0001
**Completeness of the RtHB:**						
0 recorded (none of above)		0.1 (0.1-0.2)		0.1 (0.0-0.1)		<0.0001
1 recorded		7.2 (6.7-7.8)		1.8 (1.6-2.1)		
2 recorded		29.4 (28.4-30.4)		14.7 (14.0-15.5)		
3 recorded		40.2 (39.2-41.3)		40.1 (39.0-41.1)		
4 recorded (all of above)		23.1 (22.2-24.0)		43.3 (42.3-44.4)		

CI – confidence interval, RtHB – Road to Health booklet

Completeness of the RtHB improved over time. Recording of only one or two of the four indicators decreased by 5.4 and 14.7 percentage points respectively whilst recording of all four indicators increased by 20.2 percentage points between the two survey years (*P* < 0.0001).

Having a RtHB with expected completeness (ie, all 4 indicators in 2011-12 and 2012-13), vs incomplete (including no recording of all four indicators), was then stratified by each independent variable for each survey year (**Table 3**) Overall, expected RtHB completeness increased from 23.1% in 2011-12 to 43.3% in 2012-13. Expected RtHB completeness differed significantly by age, SES and marital status for 2011-12 survey alone (**Table 3**). Overall during this survey period, between just under a fifth and a quarter of participants across age-groups, highest education achieved, marital status, parity, knowledge of MTCT, SES, infant feeding counselling, place of delivery and type of birth attendant had expected RtHB completeness. Slightly higher proportions, nearly a third, of fully complete RtHBs were observed among HIV-positive women, women who were screened for TB during pregnancy and women from the Free State, KwaZulu-Natal, Mpumalanga and Western Cape provinces. In 2012-13 expected completeness of the RtHB was also highest (at least half of the participants) among HIV-positive women, women who were screened for TB during pregnancy and women from the Free State, KwaZulu-Natal and Limpopo provinces. In most of the remainder independent variables in 2012-13, RtHBs with expected completeness ranged between 38% and 46% across sub-groups.

**Table 3:** Maternal and health provider risk factors by complete RtHB status and year in 2011-12 and 2012-13 surveys, South Africa

	2011-12, % (95% CI)		2012-13, % (95% CI)	
**Fully complete 4 indicators (N = 2465)**	Incomplete <4 indicators* (N = 7950)	**Fully complete 4 indicators (N = 3924)**	Incomplete <4 indicators* (N = 5605)
All data	23.1 (22.2-24.0)	76.9 (76.0-77.8)	43.3 (42.2-44.4)	56.7 (55.6-57.8)
**Age (years):**				
13-19	25.3 (23.1-27.7)	74.7 (72.3-76.9)	42.1 (39.2-45.0)	57.9 (55.0-60.8)
20-24	23.2 (21.6-24.9)	76.8 (75.1-78.4)	43.9 (41.9-45.9)	56.1 (54.1-58.1)
25-34	23.0 (21.6-24.5)	77.0 (75.5-78.4)	43.2 (41.5-44.9)	56.8 (55.1-58.5)
35-50	19.9 (17.6-22.4)	80.1 (77.6-82.4)	44.1 (41.0-47.3)	55.9 (52.7-59.0)
*P*-values	**0.048**		0.754	
**Mother’s education:**				
Grade 7 and below	22.8 (20.6-25.0)	77.2 (75.0-79.4)	41.6 (38.8-44.4)	58.4 (55.6-61.2)
Grade 8 and above	23.1 (22.2-24.1)	76.9 (75.9-77.8)	43.6 (42.5-44.8)	56.4 (55.2-57.5)
*P*-values	0.802		0.311	
**Marital status:**				
Single/widowed/divorced/separated	24.0 (23.0-25.1)	76.0 (74.9-77.0)	43.7 (42.5-45.0)	56.3 (55.0-57.5)
Married/co-habiting	20.3 (18.7-22.0)	79.7 (78.0-81.3)	42.0 (39.9-44.2)	58.0 (55.8-60.1)
*P*-values	**0.009**		0.325	
**Parity:**				
First child	22.7 (21.3-24.2)	77.3 (75.8-78.7)	43.0 (41.2-44.9)	57.0 (55.1-58.8)
Child 2+	23.6 (22.4-24.8)	76.4 (75.2-77.6)	44.0 (42.6-45.4)	56.0 (54.6-57.4)
*P*-values	0.425		0.458	
**Maternal reported HIV status before delivery:**				
HIV-	20.1 (19.1-21.1)	79.9 (78.9-80.9)	39.9 (38.6-41.3)	60.1 (58.7-61.4)
HIV+	31.2 (29.2-33.2)	68.8 (66.8-70.8)	52.3 (50.3-54.3)	47.7 (45.7-49.7)
*P*-values	**<0.0001**		**<0.0001**	
Correct knowledge of MTCT modes:*				
Doesn’t know MTCT/doesn’t know all modes correctly	22.2 (20.7-23.8)	77.8 (76.2-79.3)	40.8 (39.0-42.6)	59.2 (57.4-61.0)
Knows all 3 modes correctly	24.0 (22.8-25.2)	76.0 (74.8-77.2)	45.2 (43.8-46.7)	54.8 (53.3-56.2)
Response unknown	19.6 (16.8-22.6)	80.4 (77.4-83.2)	40.1 (35.6-44.8)	59.9 (55.2-64.4)
*P*-values	0.185		0.099	
**SES**				
highest	25.1 (23.3-27.0)	74.9 (73.0-76.7)	43.5 (41.3-45.8)	56.5 (54.2-58.7)
Second highest	26.3 (24.5-28.2)	73.7 (71.8-75.5)	46.1 (43.9-48.4)	53.9 (51.6-56.1)
Low	23.6 (21.8-25.4)	76.4 (74.6-78.2)	42.8 (40.7-44.9)	57.2 (55.1-59.3)
Lowest	17.5 (15.9-19.2)	82.5 (80.8-84.1)	41.1 (39.0-43.2)	58.9 (56.8-61.0)
*P*-values	**0.001**		0.291	
**TB screening during pregnancy:**				
No	18.5 (17.4-19.7)	81.5 (80.3-82.6)	39.7 (38.3-41.1)	60.3 (58.9-61.7)
Yes	31.1 (29.5-32.7)	68.9 (67.3-70.5)	50.9 (49.0-52.8)	49.1 (47.2-51.0)
Response unknown	17.6 (14.7-20.8)	82.4 (79.2-85.3)	36.9 (32.9-41.1)	63.1 (58.9-67.1)
*P*-values	**<0.0001**		**<0.0001**	
**Infant feeding counselling during pregnancy:**				
No	17.3 (14.7-20.1)	82.7 (79.9-85.3)	38.2 (34.5-42.0)	61.8 (58.0-65.5)
Yes	24.1 (23.1-25.1)	75.9 (74.9-76.9)	44.1 (43.0-45.3)	55.9 (54.7-57.0)
*P*-values	**0.008**		**0.045**	
**Place of delivery**				
Hospital	24.1 (23.1-25.2)	75.9 (74.8-76.9)	44.4 (43.2-45.7)	55.6 (54.3-56.8)
Clinic	20.2 (18.1-22.5)	79.8 (77.5-81.9)	43.0 (40.5-45.6)	57.0 (54.4-59.5)
Home/other	18.4 (14.8-22.7)	81.6 (77.3-85.2)	28.2 (23.0-34.1)	71.8 (65.9-77.0)
*P*-values	**0.015**		**0.001**	
**Birth attendant:**				
Doctor	21.4 (19.6-23.2)	78.6 (76.8-80.4)	40.8 (38.8-42.9)	59.2 (57.1-61.2)
Nurse/midwife/CHW	24.4 (23.3-25.6)	75.6 (74.4-76.7)	45.6 (44.3-47.0)	54.4 (53.0-55.7)
TBA/other	18.7 (14.8-23.2)	81.3 (76.8-85.2)	26.5 (21.1-32.7)	73.5 (67.3-78.9)
*P*-values	**0.014**		**<0.0001**	
**Province:**				
EC	21.4 (19.2-23.8)	78.6 (76.2-80.8)	22.0 (19.4-24.8)	78.0 (75.2-80.6)
FS	36.4 (33.3-39.6)	63.6 (60.4-66.7)	49.4 (46.3-52.5)	50.6 (47.5-53.7)
GP	15.5 (13.8-17.3)	84.5 (82.7-86.2)	46.2 (43.8-48.6)	53.8 (51.4-56.2)
KZN	30.5 (27.9-33.2)	69.5 (66.8-72.1)	57.5 (54.6-60.4)	42.5 (39.6-45.4)
LP	18.7 (16.5-21.1)	81.3 (78.9-83.5)	50.9 (48.1-53.7)	49.1 (46.3-51.9)
MP	34.6 (32.0-37.4)	65.4 (62.6-68.0)	46.1 (42.9-49.3)	53.9 (50.7-57.1)
NC	17.0 (14.0-20.5)	83.0 (79.5-86.0)	38.1 (33.7-42.7)	61.9 (57.3-66.3)
NW	9.0 (7.4-10.9)	91.0 (89.1-92.6)	24.8 (21.9-27.9)	75.2 (72.1-78.1)
WC	30.3 (27.9-32.8)	69.7 (67.2-72.1)	29.0 (26.5-31.6)	71.0 (68.4-73.5)
*P*-values	**<0.0001**		**<0.0001**	

CI – confidence interval, SES – socio-economic status, FS – Free State, GP – Gauteng, KZN-KwaZulu-Natal, LP – Limpopo, MP – Mpumalanga, NC - Northern Cape, NW - North West, WC - Western Cape

*Incomplete defined as 0-3 indicators.

### Summary of association between independent factors and completeness of the RtHB for 2011-12 and 2012-13 using a partial proportional odds logistic regression model

**Table 4** shows the results from the final model for testing association between predictor variables and completeness of the RtHB (expected completeness = 4 indicators recorded). Maternal age and knowledge of MTCT were not included in the final model (Wald’s test p-value >0.25). Therefore, the final model was controlled for all other remaining variables.

**Table 4:** Summary of the association between maternal and health provider risk factors and completeness of the RtHB in 2011-12 and 2012-13 surveys, South Africa; using a partial proportional odds logistic regression model

Base comparison: Completeness = 0 variables recorded	RtHB Completeness = 1 variables recorded		RtHB Completeness = 2 variables recorded		RtHB Completeness = 3 variables recorded		RtHB Completeness = 4 variables recorded	
	**Adjusted odds ratio (95% CI)**	***P*-value**	**Adjusted odds ratio (95% CI)**	***P*-value**	**Adjusted odds ratio (95% CI)**	***P*-value**	**Adjusted odds ratio (95% CI)**	***P*-value**
**Survey year ~ (ref = 2011-12):**								
2012-13	1.3 (0.3-1.7)	0.415	1.7 (1.7-1.8)	**<0.0001**	1.6 (1.6-1.7)	**<0.0001**	1.6 (1.6-1.6)	**<0.0001**
**Mother's education (ref = grade 7-):**								
Grade 8+	1.0 (1.0-1.1)	0.424	1.0 (1.0-1.1)	0.424	1.0 (1.0-1.1)	0.424	1.0 (1.0-1.1)	0.424
**Parity ~ (ref = first child)**								
Child 2+	1.0 (1.0-1.1)	0.337	1.0 (1.0-1.1)	0.337	1.0 (1.0-1.1)	0.337	1.0 (1.0-1.1)	0.337
**Marital status (ref = single/widowed/divorced/separated):**								
Married/co-habiting	0.9 (0.8-1.0)	**0.003**	0.9 (0.8-1.0)	**0.003**	0.9 (0.8-1.0)	**0.003**	0.9 (0.8-1.0)	**0.003**
**SES (ref = highest):**								**0.003**
Second highest	1.3 (0.4-4.4)	0.621	1.2 (1.0-1.5)	0.066	1.1 (1.0-1.2)	0.132	1.0 (0.9-1.1)	0.5
Low	0.5 (0.2-1.4)	0.178	1.2 (1.0-1.5)	0.117	1.1 (1.0-1.3)	**0.023**	1.0 (0.9-1.1)	0.414
Lowest	2.5(0.3-23.0)	0.408	1.1 (0.9-1.3)	0.505	0.9 (0.8-1.0)	**0.016**	0.8 (0.7-0.9)	**<0.0001**
TB screening during pregnancy* ~ (ref = No):								**0.0008**
Yes	1.6 (0.6-4.1)	0.365	1.4 (1.2-1.6)	**<0.0001**	1.5 (1.4-1.6)	**<0.0001**	1.9 (1.8-2.0)	**<0.0001**
Response unknown	0.5 (0.1-2.3)	0.364	1.6 (1.0-2.6)	**0.036**	1.2 (0.9-1.4)	0.156	0.9 (0.8-1.1)	0.505
**Infant feeding counselling during pregnancy ~ (ref = No):**								
Yes	1.6 (0.5-5.0)	0.390	2.1 (1.7-2.5)	**<0.0001**	1.6 (1.5-1.8)	**<0.0001**	1.4 (1.2-1.5)	**<0.0001**
**Place of delivery ~ (ref = home/other):**								**<0.0001**
Clinic	2.6 (1.5-3.8)	**0.001**	2.4 (1.8-3.5)	**<0.0001**	1.8 (1.4-2.2)	**0.007**	1.6 (1.3-2.1)	**0.0001**
Hospital	1.9 (1.0-3.1)	**0.008**	2.3 (1.8-3.1)	**0.003**	1.6 (1.4-2.0)	**0.0001**	1.8 (1.4-2.2)	**<0.0001**
**Birth attendant (ref = doctor):**								**0.035**
Nurse/midwife/CHW	1.3 (1.2-1.4)	**<0.0001**	1.3 (1.2-1.4)	**<0.0001**	1.3 (1.2-1.4)	**<0.0001**	1.3 (1.2-1.4)	**<0.0001**
TBA/other	1.0 (0.7-1.4)	0.957	1.0 (0.7-1.4)	0.957	1.0 (0.7-1.4)	0.957	1.0 (0.7-1.4)	0.957
**Province ~ (ref = EC):**								**0.001**
FS	0.9 (0.1-6.5)	0.920	2.5 (1.8-3.6)	**<0.0001**	3.0 (2.5-3.5)	**<0.0001**	2.6 (2.2-3.0)	**<0.0001**
GP	†	0.983	1.7 (1.3-2.2)	**<0.0001**	1.3 (1.2-1.5)	**<0.0001**	1.6 (1.4-1.9)	**<0.0001**
KZN	†	0.991	7.7(4.9-12.3)	**<0.0001**	3.1 (2.6-3.6)	**<0.0001**	2.5 (2.2-2.9)	**<0.0001**
LP	0.3 (0.1-1.8)	0.188	1.3 (1.0-1.7)	**0.032**	1.6 (1.4-1.9)	**<0.0001**	2.0 (1.7-2.3)	**<0.0001**
MP	0.7 (0.1-4.5)	0.710	1.0 (0.8-1.3)	0.786	1.3 (1.1-1.5)	**<0.0001**	2.2 (1.9-2.5)	**<0.0001**
NC	0.2 (0.0-1.3)	0.084	1.0 (0.7-1.4)	0.880	1.1 (0.9-1.3)	0.576	1.2 (1.0-1.4)	0.110
NW	0.2 (0.0-0.8)	0.029	0.5 (0.4-0.6)	**<0.0001**	0.5 (0.5-0.6)	**<0.0001**	0.6 (0.5-0.7)	**<0.0001**
WC	2.0(0.2-27.3)	0.593	10.5(6.2-7.9)	**<0.0001**	3.2 (2.8-3.8)	**<0.0001**	1.5 (1.3-1.7)	**<0.0001**

CI – confidence interval, TBA - traditional birth attendant, CHW – community health worker, SES – socio-economic status, FS – Free State, GP – Gauteng, KZN – KwaZulu-Natal, LP – Limpopo, MP – Mpumalanga, NC – Northern Cape, NW – North West, WC – Western Cape

*Category for missing data/unknown responses added.

†Frequency was zero therefore no reasonable point estimates.

Compared to having zero variables recorded on the RtHB, the odds of having all 4 variables recorded was at least 60% higher in 2012-13 compared to 2011-12 (*P* < 0.0001). Being married or co-habiting significantly reduced the odds of any level of completion in the RtHB by 10% (*P* = 0.003). Being in the lowest SES ranking also appeared to significantly reduce the odds of having 3 or all 4 indicators recorded in the booklet by 10% (*P* = 0.016) and 20% (*P* < 0.0001) respectively. Maternal education and parity were not significantly associated with completeness of the RtHB.

All four factors (TB screening during pregnancy, infant feeding counselling during pregnancy, place of delivery and birth attendant) directly related to the health care provider were significantly associated with completeness of the RtHB. The odds of having the expected completeness (all 4 indicators recorded in 2011-12 and 2012-13) of the RtHB vs zero, was significantly higher among women who had been screened for TB or received counselling about infant feeding compared to those who did not receive these services during pregnancy (all *P*-values <0.0001). Delivering at a clinic or a hospital increased the odds of expected completeness compared to delivering at home (*P*-values <0.0001). Having a nurse, midwife or community health worker handling a child birth as opposed to a doctor significantly increased the odds of having more indicators completed in the RtHB (adjusted odds ratio AOR 1.3 (95% CI = 1.2-1.4), *P*<0.0001). However, there was no difference in RtHB completeness between deliveries assisted by doctors and those assisted by traditional birth attendants. Significant differences in the completeness of the RtHB at different levels were seen across provinces. Of note were very high odds of better completeness in KwaZulu Natal (AOR≥2.5) and very low odds of better completeness (AOR≤0.6) in North West provinces compared to other provinces.

## DISCUSSION

We set out to understand how well the RtHB was used to capture four key health indicators that we used to define completeness (ie, birth weight, BCG immunisation, maternal HIV status and syphilis result) and what were the key predictors of completion. This study shows that most infants were brought to the clinic by their mothers who were mostly between 20-34 years, single, had achieved an education level of high school or more and were multiparous. This provides a unique opportunity for health care workers to engage with mothers on the development of her child as well as to educate mothers on monitoring the child’s growth and health. A study carried out at a primary, secondary and tertiary care centres in one province in South Africa showed that 85.3% of 300 carers who brought their child for care were mothers [16]. Turner and Fuller found that mothers from developing countries have a keen interest in health-related information for their children’s health and tend to search for medical information and assistance for their children more frequently than searching for other information [17]. Similarly, Senanayake et al. in a study conducted in Sri Lanka showed that mothers with a mean age of 28.6 years, education level above grade 8 and a birth order greater than three demonstrated good comprehension of their child’s growth pattern and growth chart [18] Our findings show that almost all mothers or caregivers presenting at the clinic with their infant possessed a RtHB (at least 99% in all surveys). High rates of possession of RtHB have been previously reported in studies conducted in Zimbabwe, Tanzania and India [19-21]. However, this has not been the case in earlier studies conducted in South Africa which included participants attending both well and sick child visits. Tarwa and De Villiers reported that the RtHC was not brought to 48% of the consultations [16]. Jacob and Coetzee reported a high but not satisfactory proportion of 81.3% caregivers bringing RtHBs to consultations, in a study conducted in one district in South Africa [22]. Failure to carry the RtHB may be attributed to the failure of health care workers to request for the booklet or caregivers not knowing that they have to carry the booklet to all consultations not only immunisation visits.

Our analyses demonstrate that although possession of the RtHB at the infant’s first immunisation visit is high, overall completeness of the RtHB by this time point is below 50%. In particular, recording of maternal HIV outcome (67.8% in 2012-13) and mother’s syphilis result (69.7% in 2012-13), despite gradual increase overtime, was low overall. Results from a study conducted in one province of South Africa showed that out of 109 RtHBs reviewed, only 50% had the mother’s HIV outcome indicated and PCR results were only recorded for 6% of the exposed infants [23]. Harrison et al in a study conducted in one province of South Africa also reported varying completion rates of basic birth data on the RtHC (94% birth weight recorded, 93.1% place of birth recorded, 68.7% gestational age recorded) [24].

Although reasons for poor recording of health indicators were beyond the scope of this analysis, findings from other studies have provided possible challenges for poor monitoring of the RtHB resulting in missed opportunities for immunisation or growth monitoring. Kitenge and Govender discuss several issues expressed by health care workers in monitoring the RtHB including staff shortages, lack of equipment, work overload and understaffing of nurses, stock-out of vaccines, absence of the RtHB and poor attendance of caregivers at immunisation scheduled visits [25]. Although these were not maternity-level staff, some of the challenges raised are likely to be common. Conversely, mothers expressed that health care workers rarely asked to view the RtHB during their consultation, did not plot the child’s weight on the growth chart and mothers were not informed about the development of the child [16,26]. Mothers also expressed that information on the RtHB (feeding, oral rehydration, play and stimulation and safety of the child) was not discussed with them during consultations [26].

This study was a secondary analysis from the national PMTCT surveys; therefore data explaining why the RtHB was already incomplete as early as 6 weeks post-partum were not available. Although the simplest reasons for incompleteness of the RtHB such as the caregiver not taking it along for a health care visit (user-related) or the health care provider not asking for the booklet in order to record health information (provider-related) were not evaluated directly here, our sample of mothers suggested that close to 100% of mothers had brought the card to the 6 week immunisation visit and the majority delivered with a formal health provider, but despite this the RtHBs were incomplete. This suggests the responsibility for the largely incomplete RtHBs observed here may lie primarily with provider-related factors.

Other studies conducted either in one sub-district or one province of South Africa have reported underutilization of the RtHB. These studies found that both health facility staff and mothers influence underutilization, for example, health facility staff often do not ask for the book, do not fill in the information adequately and accurately and have poor understanding and interpretation of the growth charts [16,27]. As a result they are unable to identify any deviations in the child’s growth pattern and make informed decisions on the required action [16,27]. Subsequently, mothers are not made aware of the importance of taking the RtHB to the health facility at every visit, are not educated on whether their child has received all the required immunizations, unable to interpret deviations in weight and therefore cannot recognize and react quickly when the child falls ill or does not reach developmental milestones. The outstanding (very high adjusted odds ratios) association between completeness of the RtHB and the KwaZulu-Natal and North West provinces is worth noting. The very high odds of having expected completeness in KwaZulu-Natal (**Table 4**) could mirror the better usage of RtHB among HIV-positive women (**Table 3**) because this province had very high maternal HIV prevalence of 43%-44% at the time compared to all other provinces which had less than 38% prevalence [28]. The very low odds observed for North West are however difficult to explain with the available data.

Although we could not infer causality due to the cross-sectional nature of this study, we observed that both maternal-related and health provider-related factors were associated with RtHB completeness. Being married or co-habiting and belonging to the low SES group lowered the odds of having all four indicators recorded. In the case of factors more related to the health care provider, mothers who had received TB screening or infant feeding counselling during pregnancy, had delivered at a clinic or hospital or had a nurse or midwife or a community health worker assisted delivery compared to doctor-assisted had significantly higher odds of achieving the expected completeness of the RtHB. This could reflect on the competence of the staff and/or their diligence with following general protocols and recommendations at the facilities used by mothers. The difference between doctors and nurses/midwives/community health workers is likely to reflect general responsibilities at the facilities, ie, the latter are usually the majority in primary health care services and hence likely to do more data recording duties compared to the former. This could lead to incomplete recording when doctors attend to clients without the nurses’ involvement.

The results on low recording of maternal HIV status we found here support what was being experienced in practise during the early period after the introduction of the booklet. Although no data has been published, there are unofficial reports about the PMTCT page being torn off from the booklet [29]. This was likely due to lack of privacy from other non-clinical third users (such as schools) who required basic child growth and immunization information. This could have also contributed to the low recording of the maternal HIV status variable in this data. This issue certainly needs priority attention from the South African Department of Health. It is also important to note that the indicators evaluated here are those recorded around the delivery period, hence are largely recorded by maternity-ward staff at secondary and tertiary facilities. The recording of RtHB by primary health care staff mostly begins at the 6 weeks immunization visit. It could be that the training of the use of the RtHB is not emphasized at the maternity care level, but this is a gap which needs to be investigated.

### Limitations

Our study has the following limitations. The two national evaluations were not primarily designed to evaluate the completeness of the infant RtHB within the first 6 weeks of life, therefore the data explaining why the RtHB was already incomplete as early as 6 weeks’ post-partum or the reasons for poor recording of health indicators were not available. Also, the cross-sectional nature of the study limited inference of causality on the observed associations. Infants who required emergency care at the clinic or who died prior to 4-8 weeks or who utilised mobile or private clinics or hospitals were excluded from the survey. Hence infants who received immunisations and whose RtHB was monitored at a private clinic or hospital were not part of this analysis. We do not have information which helps to answer the question of whether lack of information on the RtHB is a result of a health practitioner not asking for the booklet to record information or the caregiver did not bring the booklet. Presently, there are many health indicators that are recorded in the booklet but we were limited to those that had been evaluated in the two national PMTCT surveys done during the years 2011 to 2013.

## CONCLUSIONS

This is the first large, nationally representative study giving a good overview of the nature of use of the RtHB at the very early postnatal phase, in South Africa. Expected use of the RtHB clearly improved over time but was still unsatisfactory by 2012-13, with completeness of all four listed indicators still below 50% yet nearly all mothers brought their records to the facility. Recording of all variables requested in the RtHB, especially those introduced for PMTCT, is important for monitoring and thus preventing MTCT and improving survival. There is a need to assess the current RtHB status and see if the positive trend continued and whether steps should be taken to implement interventions for optimal usage. Since this study showed a consistent association between receipt of antenatal care TB screening, infant feeding counselling or delivery at a clinic/hospital and completeness of the RtHB, integrating promotion of the booklet with in-facility PMTCT activities could be one way to improve its completeness. It is also clear from all the findings presented in this study that interventions aiming at optimal use of the booklet need to not only focus on health providers but also on the users.
