# Supplementary material for: Completeness of patient-held records: observations of the Road-to-Health Booklet from two national facility-based surveys at 6 weeks postpartum, South Africa
Source: J Glob Health. 2018 Sep 15;8(2):020901. doi: 10.7189/jogh.08.020901 (PMC6189547; doi:10.7189/jogh.08.020901)
Supplement: Online Supplementary Document [file jogh-08-020901-s001.pdf]

Online Supplementary Document

Ramraj et al. Completeness of patient-held records: observations of the Road-to-Health Booklet from two national facility-based surveys at 6 weeks postpartum, South Africa

J Glob Health 2018;8:020901

Supplementary Table S1: Brant Test for the Parallel Regression Assumption of Proportionality of odds in the 2011-12 and 2012-13 surveys, South Africa

**Variable**	**2011-12 and 2012-13 surveys**	
	**Chi-square**	**P-value**
Survey year	40.24	<0.0001
Mother's education	0.41	0.939
Marital status	0.61	0.895
Parity	2.14	0.544
Correct knowledge of MTCT modes	~	~
SES	39.76	<0.000
TB screening during pregnancy	13.22	0.004
Infant feeding counselling during pregnancy	24.6	<0.0001
Place of delivery	27.05	<0.0001
Birth attendant	2.62	0.454
Providnce	32.02	<0.0001

Boldface - variable violates the proportional odds assumption hence not constrained in analyses. ~ not in the final mode
